# Colon carcinoma presenting as ovarian metastasis

**DOI:** 10.1016/j.radcr.2021.06.072

**Published:** 2021-07-23

**Authors:** Paul Geraeds Kemps, Mijke Bol, Ernst Johan Abraham Steller, Lisa Maria Henrica de Pont, Cynthia Holterhues, Leander van Gerven, Wendela Kolkman

**Affiliations:** a Department of Gynecology and Obstetrics, Haga Hospital, The Hague, The Netherlands; b Leiden University Medical Center, Leiden, The Netherlands; c Department of Pathology, Netherlands Cancer Institute-Antoni van Leeuwenhoek, Amsterdam, The Netherlands; d Department of Surgery, Netherlands Cancer Institute-Antoni van Leeuwenhoek, Amsterdam, The Netherlands; e Department of Radiology, Haga Hospital, The Hague, The Netherlands; f Department of Internal Medicine, Haga Hospital, The Hague, The Netherlands

**Keywords:** Adnexal mass, Ovarian metastasis, Colorectal cancer, Colon carcinoma, Krukenberg, KRAS

## Abstract

An adnexal mass is a common gynecological finding. Most adnexal masses are benign neoplasms, especially in premenopausal women. Yet, here we report a premenopausal woman with an adnexal mass that turned out to be an ovarian metastasis from colon cancer. This case emphasizes the importance of considering an ovarian metastasis in patients with (partially) solid adnexal masses and low serum CA125 levels. In addition, we identified the same *KRAS* mutation in the biopsied liver metastasis and resected right ovarian metastasis. This is in accordance with a previous molecular study of matched tumor pairs/trios of colorectal cancer patients with ovarian metastases, suggesting that mutated *KRAS* is a universal driver of the metastatic disease in women with *KRAS*-mutated colorectal cancer with ovarian metastases. More than half of all colorectal cancer patients with ovarian metastases harbor *KRAS* mutations. Future studies may investigate the efficacy of KRAS inhibitors in the treatment of these patients.

## Introduction

An adnexal mass is a common gynecological finding, and frequently detected incidentally. Most adnexal masses are benign neoplasms, especially in premenopausal women [1]. Yet, a small proportion represents malignant tumors. These malignant adnexal masses primarily comprise epithelial ovarian cancer, and more rarely ovarian metastases from secondary malignancies or uncommon primary malignancies (e.g., malignant germ cell tumors). Unfortunately, it can be difficult to differentiate between benign and malignant adnexal masses. In general, adnexal masses are not biopsied, to avoid disrupting the ovarian capsule, as this can cause dissemination of malignant cells. Hence, clinicians are reliant on clinical, radiological and laboratory parameters to determine the risk of malignancy and decide whether surgery is indicated or not. One important laboratory parameter is the serum cancer antigen 125 (CA125) level. Since it was first described in 1981, CA125 is well established as a tumor marker for epithelial ovarian cancer. Consequently, it is widely used as a diagnostic tool, supported by its incorporation in various diagnostic prediction models for ovarian malignancy [2]. However, it is crucial to remember that a low serum CA125 level does not rule out ovarian malignancy. Accordingly, here we report a premenopausal woman with a unilateral adnexal mass and low serum CA125 level that turned out to have an ovarian metastasis from colon cancer.

## Case report

**Fig 1:**
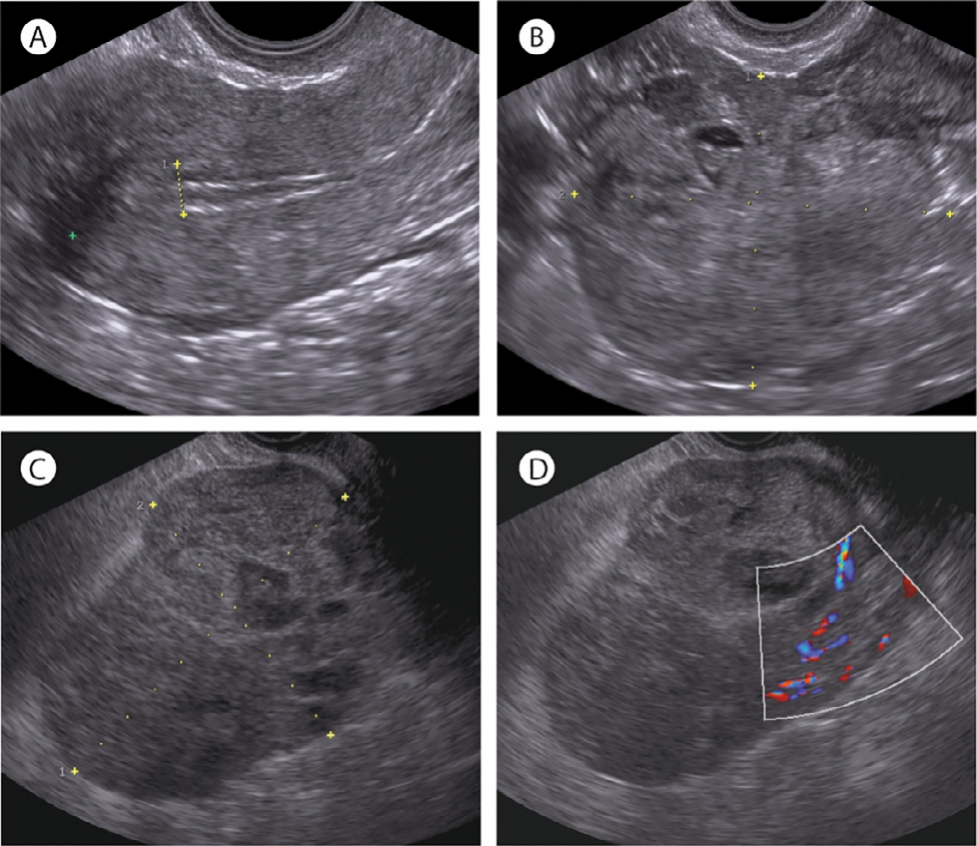
Images made during transvaginal ultrasonography at first presentation (A-B), showing a normal uterus without intracavitary abnormalities (A), and a right adnexal mass of 8×7cm (B). Images made during transvaginal ultrasonography at presentation at the gynecologic emergency unit (C-D), showing the enlarged right adnexal mass of 12×9cm with both solid and cystic components (C), and flow on color Doppler imaging (D). Yellow dotted lines indicate measurements.

**Fig. 2:**
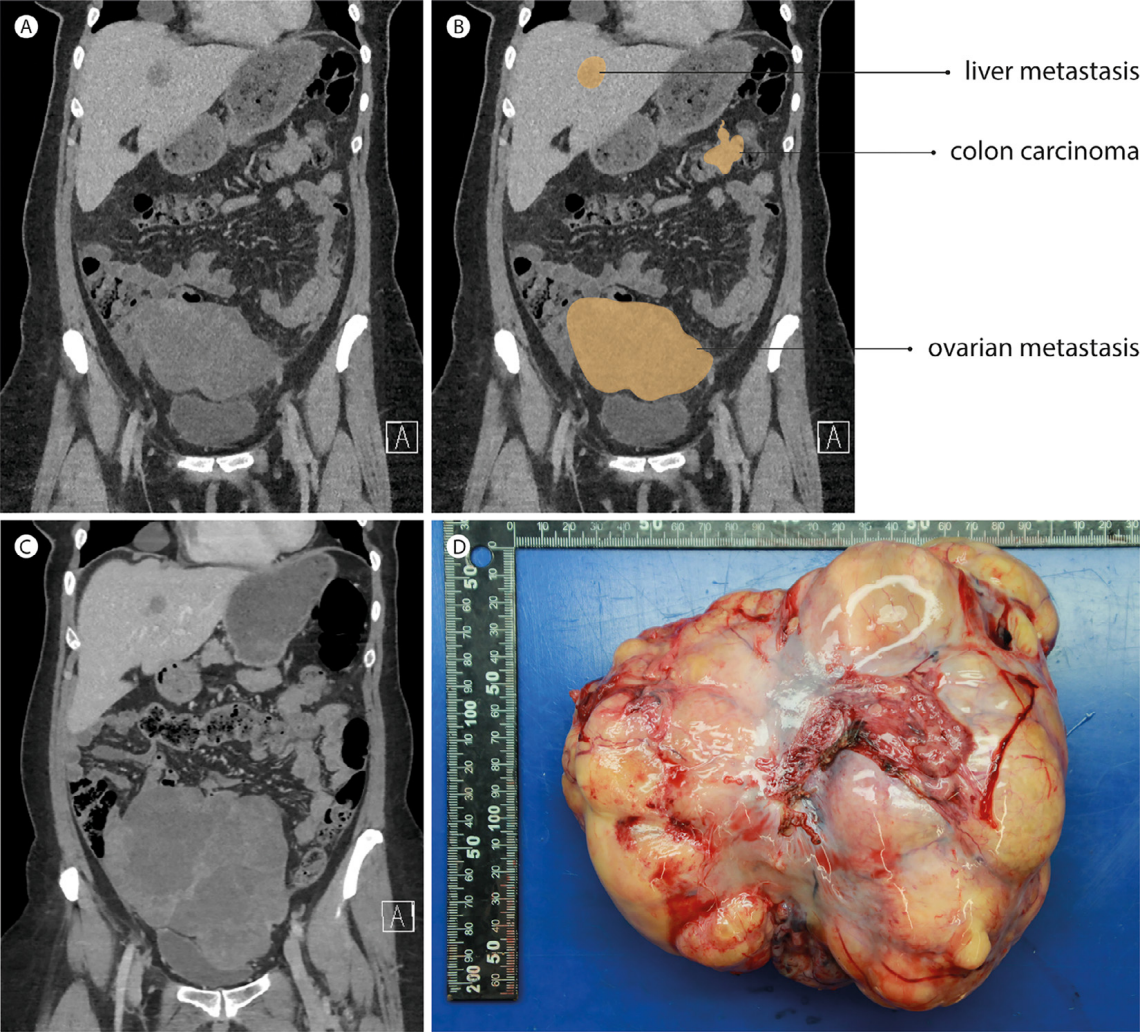
Coronal section of the contrast-enhanced abdominal CT scan performed at diagnosis (A) with graphic correlation (B), showing a 13×8cm large tumor originating from the right ovary with infiltration of surrounding mesenteric fat, as well as a mass in the distal part of the transverse colon with infiltration of mesocolic fat, and a hypodense liver lesion. The coronal section of the contrast-enhanced abdominal CT scan performed after three courses of CapOx-B and three subsequent courses of Cap-B chemotherapy (C) shows decreased size of the hypodense liver lesion (from 39 to 26mm) and increased size of the right ovarian metastasis (from 13 to 18cm). Gross macroscopic image of the resected right ovarian metastasis (D).

**Fig. 3:**
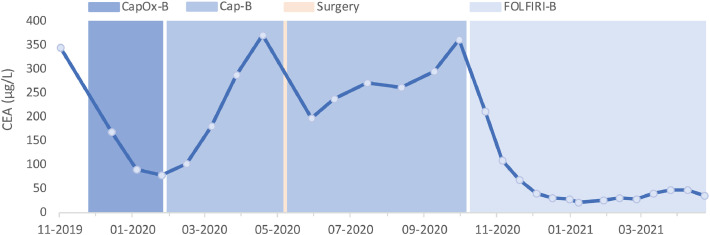
Timeline depicting the course of serum CEA levels during the period of November 2019 to May 2021. Showed are decreasing serum CEA levels during CapOx-B chemotherapy, followed by gradually increasing serum CEA levels during Cap-B chemotherapy, except for an almost 50% drop after bilateral salpingo-oophorectomy. After switch to FOLFIRI-B chemotherapy in October 2020, serum CEA levels quickly decreased again.

A 44-year-old woman presented to the gynecology clinic with menorrhagia for six months. Except for the feeling of mild pressure in the lower abdomen, she had no other symptoms. Transvaginal ultrasonography showed no uterine abnormalities (Fig. 1A), but revealed a right adnexal mass of 8×7cm with both solid and cystic components (Fig. 1B). No ascites was observed. The serum CA125 level was measured, and turned out low (18 kU/L). An abdominal computed tomography (CT) scan was requested for within two weeks. Five days later, the woman presented to the gynecologic emergency unit with increased abdominal discomfort. Transvaginal ultrasonography demonstrated significantly increased size of the right adnexal mass, which now measured 12×9cm (Fig. 1C-D). An abdominal CT scan was made the same day, revealing a right ovarian tumor, as well as a mass in the transverse colon and multiple hypodense lesions in both the right and left lobes of the liver (Fig. 2A-B). A diagnosis of metastatic colon cancer was suspected, which was subsequently supported by an increased serum carcinoembryonic antigen (CEA) level of 346 µg/L. Histopathological analysis of a liver biopsy confirmed the diagnosis, revealing classical adenocarcinoma. Immunohistochemical stainings and molecular analysis (using the Idylla^TM^ KRAS Mutation Assay) demonstrated mismatch repair proficiency and a *KRAS* p.G12V mutation, respectively. Detailed examination of the patient's family history by a clinical geneticist revealed no relatives with colorectal cancer and/or colonic polyposis. Palliative chemotherapy consisting of Capecitabine, Oxaliplatin and Bevacizumab (CapOx-B) was initiated. Oxaliplatin was discontinued after three courses due to toxicity (acute neuropathy: throat discomfort and muscle cramps). CT scans after three courses of CapOx-B and three subsequent courses of Cap-B showed gradual growth of the right ovarian metastasis (Fig. 2C), whereas the colon tumor and liver metastases had decreased in size after three courses of CapOx-B and stabilized after three courses of Cap-B. Since the abdominal discomfort had also increased, bilateral salpingo-oophorectomy was performed. Notably, Bevacizumab was discontinued several months before surgery and resumed one month after surgery to avoid impaired wound healing. In addition to the right ovarian metastasis (Fig. 2D), histopathological analysis revealed a microscopic focus of metastatic adenocarcinoma in the left ovary. Next-generation sequencing demonstrated the same *KRAS* p.G12V mutation in the right ovarian metastasis, as well as inactivating mutations in *TP53* (p.R282W) and *APC* (p.K1350fs). Chemotherapy was switched to Folinic acid, Fluorouracil, Irinotecan and Bevacizumab (FOLFIRI-B) five months after surgery due to radiological progression of the colon tumor and liver metastases, as well as increasing serum CEA levels (Fig. 3). After four courses of FOLFIRI-B, partial response was observed on abdominal CT, and serum CEA levels had significantly decreased (Fig. 3). After twelve courses, exploratory laparotomy was performed; however, colectomy and hepatic metastasectomy were not executed because multiple peritoneal metastases were observed. At eighteen months after diagnosis, the patient remains in good clinical condition (WHO performance score 0).

## Discussion

Adnexal masses may be found in women of all ages, and can represent a wide variety of lesions of the ovaries, fallopian tubes or surrounding tissues. Although most adnexal masses are benign neoplasms, many women undergo surgery due to the concern that an adnexal mass could be malignant. It is estimated that, at least in the United States, 5%-10% of all women undergo surgery for a suspected ovarian neoplasm during their lifetime [3]. However, benign adnexal masses generally do not require surgery, but can be safely managed with follow-up [4]. Moreover, there are risks associated with any surgery, with a complication rate of false-positive surgery of 3% in a recent randomized controlled trial of ovarian cancer screening in the United Kingdom [5]. Therefore, much research is aimed at discovering clinical, radiological and laboratory parameters that can be used to better determine the risk of malignancy in women presenting with an adnexal mass, so that patients can receive optimal treatment [2]. Radiologically, the proportion of solid tissue is one of the strongest predictors of malignancy [6]. Furthermore, a recent study by Landolfo *et al.* reported serum CA125 to be the single most dominant protein biomarker for discrimination of benign and malignant ovarian tumors [7]. Yet, it is important to remember that 20% of all patients with epithelial ovarian cancer have normal CA125 levels [8]. Moreover, the study by Landolfo *et al.* included only seven patients with secondary metastatic tumors, representing just 6% of 126 included patients with malignant adnexal tumors. Thus, their study mainly proved that serum CA125 is the most dominant biomarker for discrimination between benign adnexal tumors and primary ovarian malignancies, but was underpowered to investigate protein biomarkers that can discriminate between benign and secondary metastatic ovarian tumors, as well as between primary ovarian malignancies and ovarian metastases. Previous studies have reported normal serum CA125 levels in approximately 35% of patients with ovarian metastases from non-gynecological cancers [9,10]. Together with our case, these data emphasize the importance of considering an ovarian metastasis in patients with (partially) solid adnexal masses and low serum CA125 levels. In these patients, other serum biomarkers may provide preoperative clues to the metastatic origin of the adnexal mass. For example, elevated serum CEA levels may alert clinicians for the potential presence of ovarian metastases from gastro-intestinal origin [9].

Approximately 5%-15% of all malignant ovarian tumors are ovarian metastases [9]. Ovarian metastases may be the first presenting manifestation of the metastatic disease [11], as illustrated by our case. Moreover, patients can be asymptomatic or only have mild nonspecific symptoms, such as abdominal bloating or abnormal vaginal bleeding [10,11]. The colon is the most common primary malignancy site in patients with ovarian metastases, followed by the endometrium, breast, appendix and stomach [12]. Approximately half of all patients with ovarian metastases have bilateral metastases [12]. Ovarian metastases are often referred to as Krukenberg tumors, although this term is classically restricted to ovarian metastases with signet-ring cell adenocarcinoma morphology. The primary malignancy site of these “classical” Krukenberg tumors is most often the stomach, followed by the colon [12].

Ovarian metastases are present in approximately 5%-10% of all women with metastatic colorectal cancer [13]. They disproportionally affect younger women, and are associated with poor survival in comparison with national trends for metastatic colorectal cancer in general [13]. Notably, previous studies have reported frequent disproportionate growth of ovarian metastases under chemotherapy, while other sites of metastasis do respond [13], as seen in our case. Furthermore, resection of ovarian metastases has been demonstrated to be associated with significantly increased survival in patients where all other disease could also be resected [13,14]. In some of these cases, complete surgical cytoreduction may even be curative [13]. However, in patients with unresectable extra-ovarian metastases, such as our case, oophorectomy remains reserved for symptom palliation [13].

In our case, we identified the same *KRAS* p.G12V mutation in the biopsied liver metastasis and the resected right ovarian metastasis. Moreover, we identified inactivating mutations in *TP53* and *APC* in the right ovarian metastasis using next-generation sequencing. This is in line with findings of Ganesh *et al.*, who showed that *TP53, APC* and *KRAS* were the three most frequently mutated genes in tumors from 38 colorectal cancer patients with ovarian metastases. In addition, they demonstrated that *KRAS* was mutated in both the primary colorectal tumors, ovarian metastases and/or extraovarian metastases of 14/14 *KRAS*-mutated patients with available matched tumor pairs/trios [13]. These data suggest that mutated *KRAS* is a universal driver of the metastatic disease in patients with *KRAS*-mutated colorectal cancer with ovarian metastases. Interestingly, Ganesh *et al.* also reported significantly increased frequency of *KRAS* mutations in tumors of colorectal cancer patients with ovarian metastases, when compared to tumors of 426 colorectal cancer patients without ovarian metastases (66% vs 47%). Accordingly, future studies may investigate the use of KRAS inhibitors for the treatment of *KRAS*-mutated colorectal cancer patients with ovarian metastases. These novel targeted therapies are currently being tested in multiple phase I/II clinical trials [15], and recent results of the landmark phase I first-in-human trial of the KRAS p.G12C inhibitor Sotorasib showed encouraging anticancer activity in patients with heavily pretreated advanced solid tumors, including colorectal cancer [16].

### Contributions

All authors were involved in the care of the patient, and contributed to the final version of the manuscript. MB provided the gross macroscopic image of the resected right ovarian metastasis. PK prepared the figures. Written consent for publication was obtained from the patient.

## References

[1] Hermans A., Kluivers K., Janssen L., Siebers A., Wijnen M., Bulten J. (2016). Adnexal masses in children, adolescents and women of reproductive age in the Netherlands: A nationwide population-based cohort study. Gynecol Oncol.

[2] Van Calster B., Valentin L., Froyman W., Landolfo C., Ceusters J., Testa A.C. (2020). Validation of models to diagnose ovarian cancer in patients managed surgically or conservatively: multicentre cohort study. BMJ.

[3] (1994). National institutes of health consensus development conference statement. ovarian cancer: screening, treatment, and follow-up. Gynecol Oncol.

[4] Froyman W., Landolfo C., De Cock B., Wynants L., Sladkevicius P., Testa A.C. (2019). Risk of complications in patients with conservatively managed ovarian tumours (IOTA5): a 2-year interim analysis of a multicentre, prospective, cohort study. Lancet Oncol.

[5] Jacobs I.J., Menon U., Ryan A., Gentry-Maharaj A., Burnell M., Kalsi J.K. (2016). Ovarian cancer screening and mortality in the UK Collaborative Trial of Ovarian Cancer Screening (UKCTOCS): a randomised controlled trial. Lancet.

[6] Van Calster B., Van Hoorde K., Valentin L., Testa A.C., Fischerova D., Van Holsbeke C. (2014). Evaluating the risk of ovarian cancer before surgery using the ADNEX model to differentiate between benign, borderline, early and advanced stage invasive, and secondary metastatic tumours: prospective multicentre diagnostic study. BMJ.

[7] Landolfo C., Achten E.T.L., Ceusters J., Baert T., Froyman W., Heremans R. (2020). Assessment of protein biomarkers for preoperative differential diagnosis between benign and malignant ovarian tumors. Gynecol Oncol.

[8] Karam A.K., Karlan B.Y. (2010). Ovarian cancer: the duplicity of CA125 measurement. Nat Rev Clin Oncol.

[9] Stiekema A., Boldingh Q.J.A.J., Korse C.M., van der Noort V., Boot H., van Driel W.J. (2015). Serum human epididymal protein 4 (HE4) as biomarker for the differentiation between epithelial ovarian cancer and ovarian metastases of gastrointestinal origin. Gynecol Oncol.

[10] Zhang J.-J., Cao D.-Y., Yang J.-X., Shen K. (2020). Ovarian metastasis from nongynecologic primary sites: a retrospective analysis of 177 cases and 13-year experience. J Ovarian Res.

[11] Lewis M.R., Deavers M.T., Silva E.G., Malpica A. (2006). Ovarian involvement by metastatic colorectal adenocarcinoma. Am J Surg Pathol.

[12] Bruls J., Simons M., Overbeek L., Bulten J., Massuger L., Nagtegaal I. (2015). A national population-based study provides insight in the origin of malignancies metastatic to the ovary. Virchows Arch.

[13] Ganesh K., Shah R.H., Vakiani E., Nash G.M., Skottowe H.P., Yaeger R. (2017). Clinical and genetic determinants of ovarian metastases from colorectal cancer. Cancer.

[14] McCormick C.C., Giuntoli R.L., Gardner G.J., Schulick R.D., Judson K., Ronnett B.M. (2007). The role of cytoreductive surgery for colon cancer metastatic to the ovary. Gynecol Oncol.

[15] Moore A.R., Rosenberg S.C., McCormick F., Malek S. (2020). RAS-targeted therapies: is the undruggable drugged?. Nat Rev Drug Discov.

[16] Hong D.S., Fakih M.G., Strickler J.H., Desai J., Durm G.A., Shapiro G.I. (2020). KRAS ^G12C^ Inhibition with Sotorasib in Advanced Solid Tumors. N Engl J Med.

